# Diminished choroidal blood flow in hypertensive and preeclamptic third trimester pregnancies using optical coherence tomography angiography

**DOI:** 10.1371/journal.pone.0285884

**Published:** 2023-05-18

**Authors:** Alaa E. Fayed, Mohamed M. Thabet, Marwa Metwally Salama, Malak El Shazly

**Affiliations:** 1 Department of Ophthalmology, Kasr Al-Ainy School of Medicine, Cairo University, Giza, Egypt; 2 Watany Research & Development Center, Watany Eye Hospital, Cairo, Egypt; 3 Department of Obstetrics and Gynecology, Kasr Al-Ainy School of Medicine, Cairo University, Giza, Egypt; National Eye Institute, UNITED STATES

## Abstract

**Purpose:**

The aim of this study was to compare choroidal adjusted flow index (AFI) in healthy, hypertensive & preeclamptic pregnancies using optical coherence tomography angiography (OCTA).

**Methods:**

In this prospective study, healthy, hypertensive & preeclamptic third trimester pregnant women underwent OCTA imaging. 3x3 & 6x6 mm choriocapillaris slabs were exported and the parafoveal area was marked by two concentric ETDRS circles at 1 & 3 mm, centered on the foveal avascular zone. Parafoveal AFI was calculated as a parameter of choroidal blood flow.

**Results:**

Fifteen eyes of fifteen women per group were recruited (45 eyes). AFI was significantly lower in the preeclamptic compared to the healthy & hypertensive groups (Tukey HSD: <0.001 in both groups on 3x3 mm, and 0.02 & 0.04 in 6x6 mm scans), and in the hypertensive compared to the healthy group (0.005 & 0.03 in 3x3 & 6x6 mm scans respectively).

**Conclusions:**

Pregnancies complicated with preeclampsia revealed the lowest choroidal blood flow on OCTA followed by pregnancies with systemic hypertension compared to healthy pregnancies. We provide in-vivo documentation of choroidal ischemia, highlighting its culpability in hypertensive and preeclamptic retinochoroidal pathology, and the possibility of utilizing choroidal blood flow on OCTA as a precursor for disease progression.

## Introduction

Preeclampsia, commonly a disorder affecting first pregnancies, is characterized by hypertension (more than 140 mmHg systolic or 90 mmHg diastolic) occurring after 20 weeks’ gestation and may be accompanied by proteinuria [1]. A diagnosis of preeclampsia is considered when new-onset hypertension is associated with renal insufficiency, pulmonary edema, liver function abnormalities, thrombocytopenia, or visual or cerebral abnormalities [2].

Retinal and choroidal affection is considered one of the most serious ocular complications of preeclampsia, speculated to be brought on arteriolar constriction, in up to 60% of preeclamptic patients in one study [3], leading to choroidal vasogenic edema, damage of the choroidal endothelium, and eventually fibrinoid necrosis of the choroidal arterioles with delayed perfusion of the choriocapillaris [3, 4]. This process eventually leads to ischemic degeneration of the overlying retinal pigment epithelium (RPE), and a breakdown of the outer blood–retinal barrier. RPE detachments and exudative retinal detachments can develop [4].

Previous attempts at describing the underlying pathology of this retinal and choroidal aggravation in preeclamptic patients have mostly been limited to post-mortem histological studies, as well as in-vivo cross-sectional examination using optical coherence tomography (OCT) [5–7]. Despite the insightful findings of these studies, post-mortem slides fail to demonstrate the dynamic vascular changes affecting living retinal and choroidal tissue, while OCT mostly relies on extrapolating retinal and choroidal vascular changes through assessing their respective thickness, and not actual blood flow fluctuations. This has led to various contradictory revelations on these important parameters [6–8].

In recent years, optical coherence tomography angiography (OCTA) has emerged as a non-invasive tool, capable of providing a highly-detailed, cross-sectioned angiographic representation of the retinal capillary plexuses and choroidal vasculature, along with the ability to assess their perfusion state, both qualitatively and quantitatively [9–12]. These attributes can prove to be particularly advantageous in potentially more vulnerable patients like pregnant women as it allows an accurate representation of the retinal and choroidal vasculature without the need for invasive and potentially harmful dye-based imaging modalities [13].

The purpose of our study was to investigate the impact of hypertension and preeclampsia on choroidal blood flow in pregnant women in their third trimester, using OCTA, and to compare these changes with healthy pregnant women.

## Materials and methods

This was a prospective, observational, cross sectional, comparative analysis of pregnant women undergoing their routine prenatal care at the obstetrics clinic at Cairo University Hospitals in Cairo, Egypt. The study was approved by the institutional review board of Kasr Al-Ainy School of Medicine, and the ethical committees of both the departments of Ophthalmology & Obstetrics and Gynecology. The study followed the tenets of the Declaration of Helsinki and was performed in accordance with Health Insurance Portability and Accountability Act regulations. Written informed consent was obtained from all participants.

### Study sample

All pregnant women presenting to the obstetrics clinic were screened for hypertensive disorders in every antenatal care visit. Blood pressure measurement was done by a trained specialized nurse and hypertension was diagnosed clinically when systolic blood pressure was ≥ 140 mmHg or diastolic blood pressure was ≥ 90 mmHg (defined by Korotkoff phase V) occurring in two occasions at least 4 hours apart. Severe hypertension was diagnosed when systolic blood pressure ≥ 160 mmHg or diastolic pressure ≥ 110 mmHg [14]. Proteinuria screening was done using an automated dipstick. In cases showing significant proteinuria (a dipstick reading of 1+), confirmatory protein creatinine ratio was done (≥ 0.3 mg/dL). Hypertensive disorders with pregnancy were classified according to our local facility protocol shown in Table 1.

**Table 1:** Classification of hypertensive disorders with pregnancy.

Chronic hypertension	Hypertension prior to 20 weeks of gestation. No proteinuria.
Gestational hypertension	Hypertension diagnosed after 20 weeks of gestation and resolves postpartum. No proteinuria.
Preeclampsia	Gestational hypertension associated with new onset findings of one or more of the following: • Significant proteinuria^(a)^ • Thrombocytopenia^(b)^ • Renal insufficiency • Liver function impairment • Pulmonary edema • Cerebral or visual symptoms
Preeclampsia superimposed on chronic hypertension	Patient with known chronic hypertension suffering worsening of blood pressure and new onset proteinuria after 20 weeks of gestation.
Eclampsia	Preeclampsia complicated with seizure (after exclusion of other possible causes of seizures).

(a) proteinuria: ≥ 300 mg or more per 24-hour urine collection or protein/creatinine ratio of ≥ 0.3 mg/dL.

(b) Thrombocytopenia: Platelet count less than 100,000 X 10^9^/L

Note: The above-mentioned classification is used in Cairo University teaching hospital & is adopted from the American College of Obstetricians & Gynecologists (ACOG) practice bulletin [1].

Inclusion criteria were treatment-naïve eyes of healthy third trimesteric pregnant women, third trimesteric hypertensive patients & third trimesteric preeclamptic patients, based on the above-mentioned criteria. Only eyes with no clinically abnormal retinal or choroidal features were considered in order to avoid imaging artifacts, and to allow the assessment of subclinical choroidal changes in these eyes. Patients underwent OCTA imaging, as described later, and the eye with the higher quality index (Q) was used for analysis. When both eyes showed a similar Q index, the right eye was uniformly chosen. For all patients, only eyes that had OCTA images without significant movement or shadow artifacts, a Q index of 6 or more and a signal strength index (SSI) score above 50 were considered eligible.

Exclusion criteria included patients with any other systemic disorders which may affect the retinochoroidal vasculature, including diabetes mellitus, renal, cardiovascular, or immunological disease. They also included eyes with other retinal or choroidal diseases that may confound our results, eyes with vitreomacular traction causing macular edema, and eyes that have received intravitreal anti-VEGF agents, intravitreal triamcinolone acetonide, dexamethasone intravitreal implant, focal laser or pars plana vitrectomy. We excluded eyes with astigmatism (more than 3 diopters), high refractive error (more than 6 diopters), or cataract graded above nuclear opalescence grade three or nuclear color grade three, to avoid optical artifacts that may potentially compromise OCTA image quality. Electronic medical records were reviewed to extract demographic and clinical information.

### OCT angiographic imaging and segmentation

Patients underwent imaging using RTVue-XR Avanti device (Optovue Inc, Fremont, California, USA), with split-spectrum amplitude-decorrelation angiography (SSADA) software [15]. This instrument has an A-scan rate of 70,000 scans per second and uses a light source centered at 840 nm and a bandwidth of 45 nm. Two consecutive B-scans (M-B frames), each containing 304 A-scans, were captured at each sampling location and SSADA was used to extract OCTA information. 3D Projection artifact removal (3D-PAR) technology by Optovue was used to obtain 3x3 & 6x6 mm scans centered on the fovea [16]. *En face* OCT angiograms were segmented automatically using the built-in software to define the choriocapillaris. The inner boundary of the *en face* image segment was set at 10 μm above, and the lower boundary at 30 μm below the Bruch’s membrane. The angiograms were then exported for adjusted flow index analysis.

### Adjusted flow index (AFI) calculation

**Fig 1:**
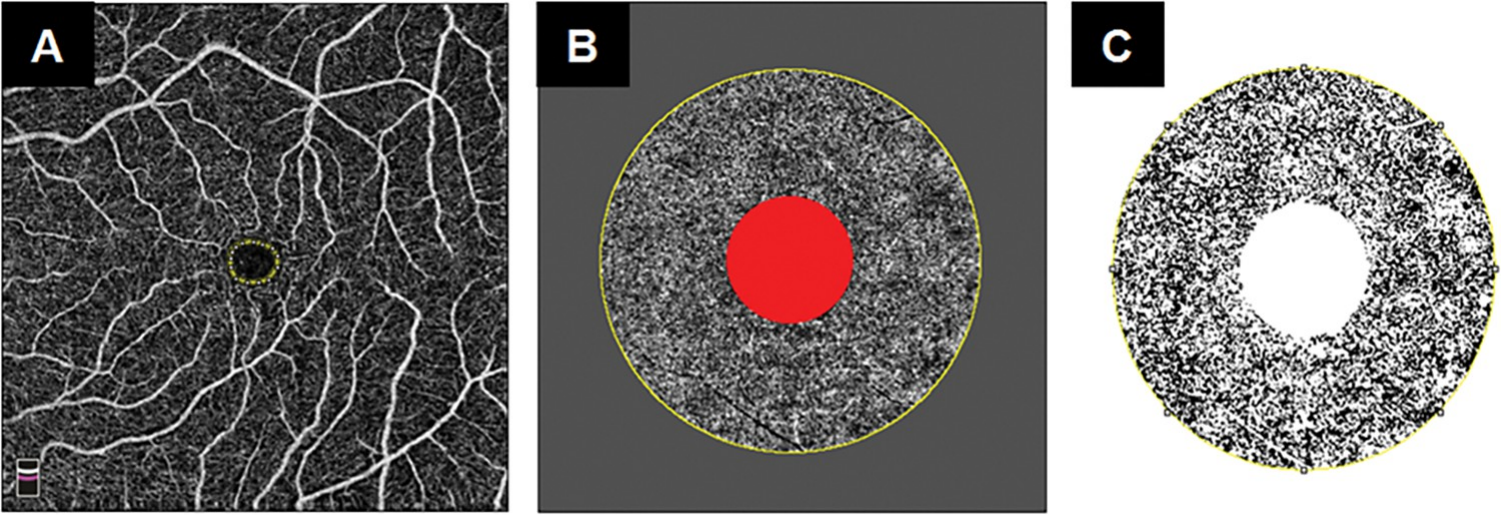
Parafoveal adjusted flow index calculation. (A) 6x6 mm^2^ full retinal thickness OCTA slab with a yellow circle delineating the foveal avascular zone used to establish the noise threshold. (B) Choriocapillaris OCTA slab fitted with1- & 3-mm circles centered on the FAZ to outline the parafovea. (C) Binarized vessels of the choriocapillaris parafovea with elimination of pixels below the threshold. Average pixel intensity above the threshold was used to calculate adjusted flow index (AFI).

We exported the full retinal thickness & choriocapillaris angiograms into ImageJ [17] (National Institutes of Health [NIH], Bethesda, MD, USA). We started by establishing a global threshold for each eye to distinguish vessels from background noise. On the full retinal thickness angiogram, we selected an area within the foveal avascular zone (FAZ) using a circle outlining its boundaries. We obtained the mean pixel intensity within the circle and repeated this three times. We took the average of the three selections as the noise level for that eye. All pixels with intensities above the noise level were considered ‘vessels’. The parafovea was marked by creating 1- and 3-mm circles, centered on the anatomical center of the foveal avascular zone (FAZ) of the corresponding full retinal thickness slab. The foveal area subtended by the 1 mm circle and the perifoveal area outside the 3 mm circle were excluded, and only the parafoveal area subtended between the two circles was used for analysis (Fig 1). We decided to utilize the parafoveal area instead of the whole slab in order to ensure the same anatomical portion of the choriocapillaris is compared in all study subjects& eliminate the risk of eccentric image fixation. We calculated the parafoveal AFI, which is defined as the average decorrelation value of all pixels above the noise threshold (only ‘‘vessels”) in the *en face* angiogram of the parafoveal area [15, 18].

To eliminate the risk of bias, the images were randomized, and the grader was masked to the randomization process. The figures were calculated twice and an intra-class correlation coefficient (ICC) for the repeatability of the assessed parameter was recorded.

### Statistical analysis

We used IBM SPSS statistics version 25 (IBM SPSS Statistics; IBM Corporation, Chicago, IL, USA). Intra-class correlation coefficient (ICC) for the repeatability of the measurement of adjusted flow index by the single grader (intra-grader repeatability) was 0.936. Shapiro-Wilk tests were used to determine if data was distributed normally. One-way analysis of variance (ANOVA)was used to examine the global difference between the three groups in terms of age, as well as adjusted flow index. Post-hoc Tukey Honest Significant Difference (HSD) analyses were run between group pairs. A p value of <0.05 was considered statistically significant.

Sample size was calculated using Power Analysis and Sample Size (PASS) Software (version 15, NCSS, LLC. Kaysville, Utah, USA.) Based on a worldwide prevalence of preeclampsia at an estimated 2% [19], a sample size of 13 eyes of patients with preeclampsia achieves 80% power at a 0.05 significance level. We increased this number to 15 eyes to account for potential image quality difficulties, in addition to 15 eyes of healthy pregnancies, and another 15 eyes with hypertensive pregnancies showing no preeclampsia.

## Results

The study included 15 eyes of 15 patients in each of the three study groups: healthy third trimester, hypertensive third trimester & preeclamptic third trimester patients, for a total of 45 eyes of 45 patients. Consecutive sampling of pregnant women attending the OBGYN clinic who met the inclusion and exclusion criteria was followed. The overall demographics and disease-related characteristics are reported in Table 2.

**Table 2:** Demographic, disease-related and image-related patient characteristics.

		*Healthy controls*	*Hypertensive*	*Preeclamptic*	*p-value*
**Patients (n)**		15	15	15	
**Age; years (mean ± SD)**		24.8 ± 3.4	26 ± 4.2	26.3 ± 4.4	0.382
**Parity (mean ± SD)**		2 ± 1.5	2.2 ± 1.7	1.9 ± 1.4	0.374
**ABP (mean ± SD)**	**Systolic**	116.6 ± 6.9	153.3 ± 11.6	164 ± 15.8	0.068
	**Diastolic**	75.6 ± 5.6	92.6 ± 6.2	105.6 ± 8.3	0.061
**Refractive error (D) (mean ± SD)**		-0.6± 2.5	+0.35± 1.75	+0.75 ± 2.25	0.335
**Image quality value (mean ± SD)**		8.4±0.25	8.5±0.20	8.5 + 0.20	0.275

SD: Standard deviation, ABP: Arterial blood pressure, D: Diopters.

**Fig 2:**
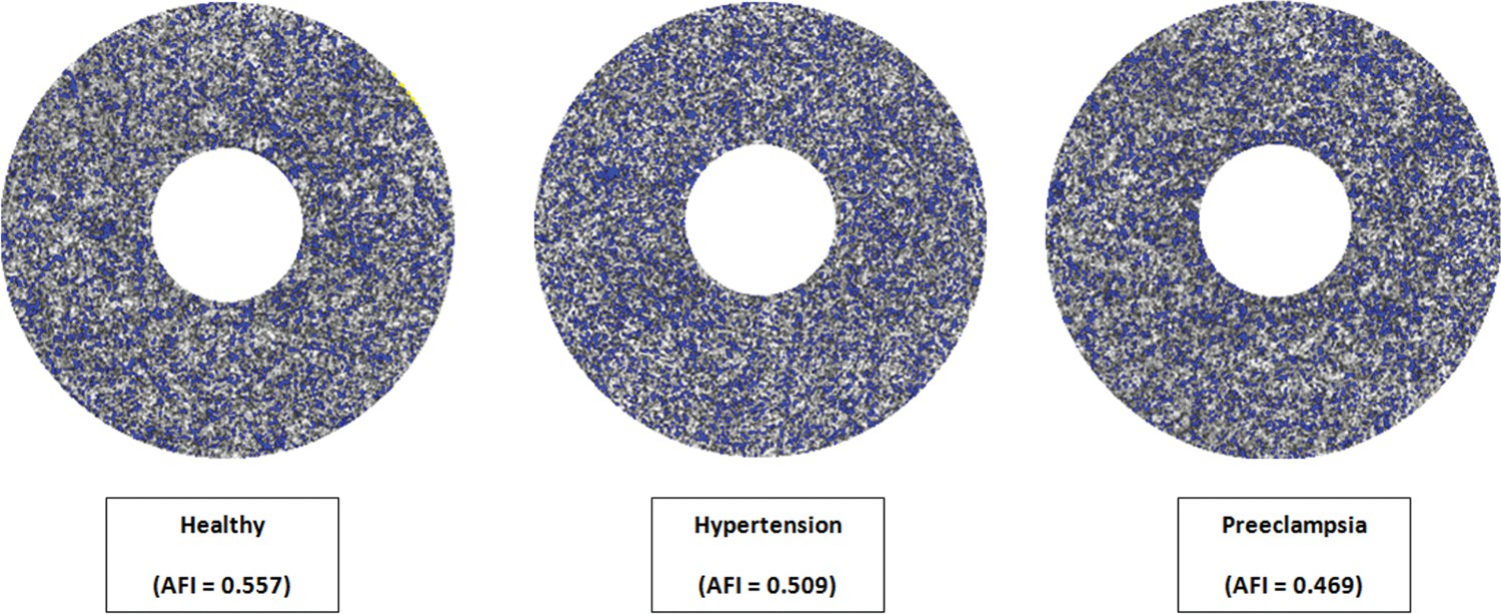
Parafoveal adjusted flow index calculated in 3x3 mm scans of 3 eyes from each of the study groups.

In both 3x3 & 6x6 mm scans, a statistically significant difference in the parafoveal choroidal AFI between the three study groups: healthy third trimester, hypertensive third trimester & preeclamptic third trimester, has been recorded. (ANOVA p value: <0.001 in the 3x3 mm scans & 0.03 in the 6x6 mm scans). (Fig 2) AFI was significantly lower in the preeclamptic group compared to both the healthy & hypertensive groups (Tukey HSD p value: <0.001 in both groups on the 3x3 mm scans, and 0.02 & 0.04 respectively in the 6x6 mm scans), as well as in the hypertensive group compared to the healthy group (Tukey HSD p value: 0.005 & 0.03 in the 3x3 mm & 6x6 mm scans, respectively). The detailed results can be found in Table 3.

**Table 3:** Recorded parafoveal choroidal adjusted flow index (AFI) in the three study groups.

	*Healthy*	*HTN*	*Preeclampsia*	*Overall ANOVA p value*	*Pairwise comparison*			
					*Healthy vs*. *HTN*		*HTN vs*. *Preeclampsia*	*Healthy vs*. *Preeclampsia*
**3x3 macular scans (mean ± SD, Range)**	0.526 ±0.021 (0.494–0.572)	0.504 ±0.022 (0.453–0.531)	0.476 ±0.019 (0.441–0.51)	**<0.001**	**0.005**	**<0.001**		**<0.001**
**6x6 macular scans (mean ± SD, Range)**	0.515±0.06 (0.439–0.636)	0.482 ±0.031 (0.418–0.529)	0.477 ±0.023 (0.437–0.51)	**0.03**	**0.04**	**0.04**		**0.02**

HTN: Hypertension, 3x3: 3x3 mm, 6x6: 6x6 mm, SD: Standard deviation, vs.: versus

## Discussion

In this study, we used both 3x3 & 6x6 mm OCTA macular scans to evaluate the variations in adjusted blood flow in the subfoveal choriocapillaris slab in healthy, hypertensive & preeclamptic third trimester pregnancies. We demonstrated a statistically significant difference in the parafoveal choroidal blood flow between the three groups, where healthy individuals showed significantly higher blood flow compared to both hypertensive & preeclamptic patients, and the same trend was found comparing hypertensive patients to those presenting with preeclampsia.

Pregnancy is associated with significant vascular changes affecting most organs & systems, attributed mostly to the hormonal variations occurring with pregnancy. Although the mainstay of these changes is vasodilation & early hypotension brought on by various prostaglandins & endothelium-dependent factors, some pregnancies are complicated by elevated levels of blood pressure, ie. gestational hypertension, which may materialize into preeclampsia, characterized by urinary loss of protein, or more life-threatening conditions, such as eclampsia & HELLP syndrome [20].

The impact of hypertension, both in the setting of pregnant and non-pregnant individuals, on the ocular circulation has been extensively stated in literature. Nevertheless, the study of the retinal & choroidal changes brought on by hypertension during pregnancy have largely been limited by the lack of adequate angiographic representation of these changes based on the inability to utilize dye-based imaging modalities, such as fluorescein & indocyanine green angiography, which are speculated to be hazardous to the growing fetus [21]. These circumstances have mostly limited imaging options to other less informative, yet safer, modalities like OCT & doppler velocimetry [22–25]. Despite the insight these options provide, their inability to provide representations of the retinal & choroidal circulations continued to hinder our understanding of these changes, and the possibility of predicting their outcomes.

Considered one of the most promising imaging modalities in overcoming these mentioned setbacks, OCTA is capable of presenting high quality angiographic representations of the retinal and choroidal vascular network, which are superimposed on the structural cross section of the OCT [10, 26]. It also provides unique *en face* depictions of the vascular anatomy of the retina and choroid, along with the capacity to modulate these images based on retinal layer segmentation [11]. When applying these assets to studying gestational hypertension & preeclampsia, it offers the a safe, convenient, non-invasive & dye-less alternative that that is easily repeated for the frequent follow-up of these eyes.

Several studies were able to demonstrate significantly decreased levels of macular capillary vessel density & retinal blood flow in eyes of poorly controlled hypertensive patients compared to healthy individuals & hypertensive patients with better control of their arterial blood pressure [27–29]. These results were consistent with our findings in hypertensive pregnant individuals, which also highlight a similar reduction in the choroidal circulation, thus emphasizing the global impact of hypertension on the posterior ocular circulation, and providing a convenient tool for monitoring these changes. On the other hand, another report identified increasing choroidal flow in patients with systemic hypertension using OCTA [30]. Although these findings may seem contradictory to ours, it is important to realize that there is a difference in the studied subjects. A recent report described lower choroidal blood flow in eyes of healthy pregnant women compared to non-pregnant healthy counterparts [31]. The authors attributed these findings to a potential impact of pregnancy-related hormonal changes. It is possible that these hormonal changes and the potentially “lower baseline” choroidal blood flow in pregnant women may potentially lead to a different response to elevated blood pressure than those identified in non-pregnant hypertensive individuals. In addition, the rapidly elevated gestational blood pressure may elicit more of a reflex vasoconstrictive effect than in chronically elevated non-gestational hypertension, which may not respond as dramatically.

Pertaining to preeclampsia, similar findings to those detected in hypertension have been observed. Both the superficial and deep foveal & parafoveal densities were recorded to be significantly lower in preeclamptic women compared to healthy pregnant & non-pregnant individuals [32, 33]. Hata et al. [34] documented diminished pulsatility in the ophthalmic artery in preeclamptic patients compared to healthy pregnant women using Doppler imaging. Once again, these declining trends in retinal tissue perfusion echo the changes found in the choroidal circulation in our study groups. Interestingly, our results demonstrate a more enlightening comparison between choroidal blood flow in preeclamptic & hypertensive patients. The significantly lower choroidal perfusion in preeclamptic eyes suggest a subtle threshold of choroidal ischemia exists, above which the level of tissue deoxygenation may be too drastic for the retinal pigment epithelium to tolerate, paving the way to the various retinal & choroidal change seen in severe cases of preeclampsia [35]. This theory can be validated by the improving ischemic foci & choroidal reperfusion demonstrated by Saito et al. [36] in eyes recovering from hypertensive choroidopathy. In addition, one may argue that OCTA provides several advantages over doppler assessment including the lack of need for significant operator experience, and the possibility of dissociating choroidal from retinal changes, where the most clinically relevant deleterious effects of elevated blood pressure seem to occur.

Our study was limited by the relatively small number of eyes, which was imposed by our use of strict criteria to ensure the quality of the included images, as well as the delicate & vulnerable nature of our study groups. We also did not include analysis of the retinal blood flow & capillary density indices, in order to focus on the novelty of the choroidal perfusion indices that are widely believed to be the culprit behind the ischemic changes seen in gestational hypertensive & preeclamptic patients. Moreover, we did not perform a longitudinal analysis of these changes after the termination of pregnancy, which can be the prospect of a future study to explore the reversibility of these choroidal flow changes. Lastly, we did not use swept-source OCTA to explore whether these results would be extrapolated when using a longer wavelength, which may theoretically exhibit less artifactual images of the choroid. The strengths of our study include the inclusion of both hypertensive & preeclamptic study groups, rather than only preeclamptic patients, in order to underscore the existing differences in choroidal blood flow between the two disease entities & the possibility of utilizing them as a precursor for disease progression.

## Conclusions

In summary, our study used 3x3 & 6x6 mm macular OCTA scans to demonstrate significantly lower parafoveal choroidal blood flow in third trimester pregnant women with preeclampsia & systemic hypertension compared to healthy third trimesteric pregnant women. We also highlighted lower choroidal blood flow in preeclampsia compared to pregnancy-related hypertension. These findings provide insight into the impact of increasingly elevated levels of arterial blood pressure on the choroidal circulation & their potential burden on the function of the overlying retinal pigmentary pump mechanism. Larger longitudinal studies are needed to examine the possibility of utilizing choroidal blood flow on OCTA as a potential marker for follow up of pregnant women with warning signs of developing gestational hypertension & preeclampsia. These findings would carry significant implications on the potential role of choroidal blood flow assessment as a routine non-invasive prenatal care modality capable of providing diagnostic data for preeclampsia from other less threatening hypertensive disorders.
